# Perfil Clínico, Autonômico e Escore de Calgary Modificado de Crianças e Adolescentes com Presumida Síncope Vasovagal Submetidos ao Teste de Inclinação

**DOI:** 10.36660/abc.20220543

**Published:** 2023-07-27

**Authors:** Pamela Michelle Leite Oliveira, Rose Mary Ferreira Lisboa da Silva, Henrique de Assis Fonseca Tonelli, Zilda Maria Alves Meira, Cleonice de Carvalho Coelho Mota

**Affiliations:** 1 Universidade Federal de Minas Gerais Belo Horizonte MG Brasil Universidade Federal de Minas Gerais, Belo Horizonte, MG – Brasil; 2 Hospital das Clínicas Universidade Federal de Minas Gerais Belo Horizonte MG Brasil Hospital das Clínicas da Universidade Federal de Minas Gerais, Belo Horizonte, MG – Brasil

**Keywords:** Síncope Vasovagal, Frequência Cardíaca, Teste da Mesa Inclinada, Criança, Adolescente

## Abstract

**Fundamento:**

A síncope, na população pediátrica, tem como sua principal causa, a vasovagal (SVV). Sua avaliação deve ser feita por métodos clínicos e o teste de inclinação (TI) pode contribuir para seu diagnóstico.

**Objetivos:**

Analisar o perfil clínico, os escores de Calgary e de Calgary modificado, a resposta ao TI e a variabilidade da frequência cardíaca (VFC) de pacientes ≤ 18 anos de idade, com presumida SVV. Comparar as variáveis entre pacientes com resposta positiva e negativa ao TI.

**Método:**

Estudo observacional e prospectivo, com 73 pacientes com idades entre 6 e 18 anos, submetidos à avaliação clínica e ao cálculo dos escores, sem o conhecimento do TI. Este foi feito a 70º sob monitoramento para análise da VFC. Valor-p < 0,05 foi considerado como o critério de significância estatística.

**Resultados:**

A mediana de idade foi de 14,0 anos, sendo que 52% eram no sexo feminino, 72 apresentaram Calgary ≥ -2 (média 1,80) e 69 com Calgary modificado ≥ -3 (média 1,38). Ocorreram pródromos em 59 pacientes, recorrência em 50 e trauma em 19. A resposta ao TI foi positiva em 54 (49 vasovagal, com 39 vasodepressora), com aumento do componente de baixa frequência (BF) e diminuição da alta frequência (AF) (p < 0,0001). Na posição supina, o BF foi de 33,6 no sexo feminino e 47,4 em unidades normalizadas no sexo masculino (p = 0,02). Aplicando-se a curva de operação característica para TI positivo, não houve significância estatística para VFC e os escores.

**Conclusões:**

A maioria das crianças e adolescentes com diagnóstico presumido de SVV apresentaram um cenário clínico típico, com escore de Calgary ≥ -2, e resposta vasodepressora predominante ao TI. Verificou-se uma maior ativação simpática na posição supina no sexo masculino. Os escores de Calgary e a ativação simpática não permitiram predizer a resposta ao TI.

## Introdução

A síncope é caracterizada pela perda transitória da consciência e do tônus postural, resultante da hipoperfusão cerebral transitória. Apresenta início repentino, pequena duração e recuperação espontânea e completa.^1 , 2^ A síncope pediátrica tem inúmeras causas, no entanto, a principal delas, em cerca de 80% dos casos, é a vasovagal (SVV).^3^

A avaliação de síncope, em sua maioria, é feita pela anamnese; exame físico; aferição de pressão arterial (PA), em posição supina e ortostática; e realização de eletrocardiograma (ECG).^1 , 2^ Um instrumento essencial para a propedêutica de síncope em crianças e adolescentes é o escore de Calgary modificado, utilizado para realizar o diagnóstico diferencial em pacientes com SVV e demais causas de síncope.^4^

Após uma abordagem clínica adequada, o teste de inclinação (TI) é uma ferramenta valiosa e pode ser utilizada, evitando exames laboratoriais desnecessários, em pacientes sem indícios de cardiopatia e com história clínica sugestiva de SVV, nos quais esse diagnóstico ainda não tenha sido realizado e/ou seja necessária a elucidação do padrão de resposta ao TI.^5^ Na população pediátrica, sua sensibilidade e especificidade podem ser de 76,6% e 86,7%, respectivamente.^6^ O TI contribui para o diagnóstico diferencial entre SVV e seus subtipos: vasodepressora, cardioinibitória, mista, síndrome de taquicardia postural ortostática (STPO) e hipotensão ortostática (HO), assim como na distinção entre síncope e epilepsia.

Sabe-se que os distúrbios do sistema nervoso autônomo (SNA) estão implicados na fisiopatologia da SVV. Pacientes com essa condição apresentam atividades basais simpática aumentada e parassimpática diminuída, em relação a indivíduos saudáveis, o que pode ser evidenciado sob condições provocativas, como durante o TI.^7^ Assim, a avaliação do SNA é uma ferramenta importante na compreensão da síncope e na condução da propedêutica desses pacientes.

Em razão da escassa literatura sobre SVV, TI e avaliação do SNA na população pediátrica, este trabalho teve como objetivos analisar o perfil clínico, os escores Calgary e Calgary modificado; sua associação com a ocorrência de pré-síncope ou síncope ao TI em crianças e adolescentes com história de SVV; e avaliar a variabilidade da frequência cardíaca (VFC) pela análise espectral antes do TI, na posição supina, e durante o TI.

## Métodos

Tratou-se de um estudo de coorte, observacional, transversal e prospectivo, realizado no período de fevereiro de 2016 a março de 2020. A pesquisa foi aprovada pelo comitê local de ética em pesquisa e os participantes e/ou seus responsáveis assinaram o termo de assentimento e/ou o termo de consentimento livre e esclarecido. A casuística foi composta por 73 pacientes. Foram incluídos aqueles com idade igual ou superior a 6 anos, com até 18 anos, com diagnóstico presumido de SVV e sem cardiopatia estrutural, assim como sem canalopatias (com ECG normal, sem histórico pessoal e/ou familiar de síncope ou morte cardíaca súbita por arritmia). Foram excluídos pacientes com doenças cardíacas, neurológicas, psiquiátricas e crônicas; pacientes com arritmias; pacientes usuários de fármacos com interferência potencial sobre a velocidade de condução do coração, frequência cardíaca (FC) ou PA, bem como aqueles em ritmo de marca-passo artificial e quaisquer outras condições que pudessem alterar o ritmo cardíaco.

Os pacientes foram submetidos à avaliação clínica e foram feitos os cálculos dos escores Calgary e Calgary modificado ( Tabela 1 ),^4 , 8^ por um pesquisador sem o conhecimento do resultado do TI. Os TIs foram realizados no turno da manhã, em uma sala adequada e sob baixa luminosidade. Os pacientes estavam em jejum de 6 horas e com acesso venoso periférico eletivo, permanecendo na posição supina em repouso durante 20 min. A mesa empregada apresentava apoio para os pés e permitia variações do ângulo de inclinação em intervalos de 10 graus. Os testes foram realizados com ângulo de 70 graus e durante o tempo limite de 30 minutos, caso não ocorresse a resposta positiva. Os pacientes foram monitorados com ECG contínuo de 12 derivações e PA não invasiva no braço (registrada a cada 3 min), desde a fase de repouso até o final da recuperação, conforme a normatização dos equipamentos e técnicas para a realização de exames do TI.^9^ Foi feito, também, o monitoramento por meio de Holter Cardiosmart para avaliação da VFC. Este foi realizado na posição supina, e nos 10 primeiros ou últimos 5 minutos do TI antes do evento sincopal, no caso de resposta positiva. A análise foi feita após correção de extrassístoles, pausas e interferências por cardiologista habitado, utilizando-se a transformação de Fourier, obtendo-se os valores absolutos de potência em milissegundos ao quadrado (ms^2^) e os valores em unidades normalizadas (un) dos componentes de baixa frequência (BF), alta frequência (AF) e a relação BF/AF. Conforme a literatura, a atividade vagal é a principal do componente AF e a relação BF/AF reflete o equilíbrio simpático-vagal.^10^

**Tabela 1:** – Itens da pontuação do escore de Calgary modificado ^4^

Questão	Pontuação (se resposta positiva)
Há bloqueio bifascicular ao ECG do paciente ou histórico de assistolia ou taquicardia supraventricular?	- 5
Os espectadores notaram, às vezes, coloração azulada no paciente durante a síncope?	- 4
Os episódios de síncope iniciaram quando o paciente apresentava a idade ≤ 5 anos?	- 3
O paciente se lembra de algo enquanto estava inconsciente?	- 2
O paciente sente que vai “desmaiar” ao ficar sentado ou em pé por muito tempo?	+ 1
Há sudorese ou sensação de calor antes da síncope?	+ 2
Há sensação de “desmaio” durante episódios de dor ou estando em áreas médicas?	+ 3

ECG: eletrocardiograma. OBS: No escore de Calgary, ^8^ as diferenças estão na questão 1 (que inclui também diabete) e na questão 3 (que considera o início dos episódios de síncope com 35 anos ou mais).

### Análise estatística

A análise estatística foi realizada por meio do programa SPSS para Windows versão 14.0 (SPSS, Inc., Chicago, Illinois). As variáveis qualitativas foram apresentadas por meio de frequências, as variáveis contínuas por meio de média e desvio padrão (para variáveis com distribuição normal), e mediana e intervalos interquartis (para variáveis sem distribuição normal), conforme o teste de Kolmogorov-Smirnov.

A comparação entre grupos foi realizada da seguinte forma: teste t de Student (não pareado) para variáveis contínuas de distribuição normal ou teste não paramétrico de Mann-Whitney para variáveis de distribuição não gaussiana; teste de qui-quadrado para variáveis qualitativas (ou teste exato de Fisher, quando pertinente). O teste de Wilcoxon foi utilizado para comparar variáveis pareadas. Foi aplicada a curva de operação característica para avaliar a sensibilidade e a especificidade dos escores de Calgary e de Calgary modificado, e dos componentes da análise espectral, considerando a resposta positiva ao TI. Valor-p < 0,05 foi considerado como o critério de significância estatística.

## Resultados

### Características gerais da casuística

A mediana da idade dos pacientes foi de 14,0 anos (intervalo interquartil – Q1-Q3: 11,0 - 16,0) variando de 6 a 18 anos, sendo 38 crianças do sexo feminino (52,0%). Cinquenta e nove pacientes apresentaram pródromos (80,8%) e 19 relacionaram a traumas físicos decorrente da SVV. Os sintomas prodrômicos mais relatados foram: astenia, palidez cutânea, sudorese fria e visão turva. Verificou-se histórico de recorrência (considerada igual ou maior do que 3 episódios de síncope) em 50 pacientes. Setenta e dois apresentaram Calgary ≥ -2, com média 1,80, e 69 apresentaram Calgary modificado ≥ -3, com média de 1,38.

A mediana do tempo de evolução dos sintomas foi de 19,0 meses (4,5 – 55,0). A mediana do intervalo entre o último episódio de síncope e o TI foi de 41,0 dias. Quanto ao número de episódios, a mediana foi de 4,0 (2,0 – 8,0).

Em relação aos exames complementares realizados antes do TI, 42 pacientes foram submetidos ao ecocardiograma, 31 ao monitoramento pelo Holter, 13 ao teste ergométrico, 36 a exames de patologia clínica, e 28 foram avaliados pela neurologia.

### Associação de variáveis clínicas

Com relação à variável sexo, houve diferença quanto à idade, com mediana de 15,0 anos (13,7 – 16,0) no sexo feminino, e de 13,0 (10,0 – 15,0) no sexo masculino, p = 0,02. Não houve associação com as variáveis de tempo de evolução, proporção de pródromos, trauma, número de episódios de síncope, intervalo entre o último episódio e o dia da realização do TI, e os escores Calgary e Calgary modificado. Ambos os sexos foram submetidos à mesma proporção de exames complementares.

Quanto à injúria física, houve associação apenas com o escore Calgary modificado, o qual apresentou a média de 0,7 no grupo com injúria física e de 1,6 no grupo sem essa injúria (p = 0,01).

### Variáveis relacionadas ao TI

A resposta ao TI foi positiva em 54 pacientes (74%), a saber: vasovagal em 49, HO em 3 e STPO em 2. No que se refere à resposta tipo vasovagal, 39 apresentaram resposta vasodepressora, 7 cardioinibitória e 3 apresentaram resposta mista. A média de tempo para resposta vasovagal ao TI foi de 13,9 ± 9,4 minutos, variando de 1,25 a 33 minutos.

A média da queda da PA sistólica durante o TI foi de 110 mmHg em pacientes com resposta vasodepressora e a média da queda na FC foi de 61 bpm em pacientes com resposta cardioinibitória. Dentre os pacientes com resposta positiva ao TI, 9 não apresentaram recuperação espontânea completa após o retorno à posição supina, sendo necessária intervenção médica com administração de atropina (dose máxima de 1,0 mg) e/ou soro fisiológico 0,9% (máximo de 300 ml).

### Avaliação da variabilidade da frequência cardíaca por meio da análise espectral

Ao comparar os componentes da VFC na posição supina e aos 10 minutos de inclinação pelo teste de Wilcoxon, em toda a casuística, foram obtidos os valores médios dos componentes BF, AF e a relação entre os dois, demonstrados na Tabela 2 .

**Tabela 2:** – Análise espectral da frequência cardíaca dos pacientes na posição supina e aos 10 minutos no teste de inclinação

Variáveis	Posição supina	10 min TI	Valor-p
BF (ms^2^)	1819,4	1326,6	0,05
BF (un)	44,0	61,6	< 0,0001
AF (ms^2^)	2735,9	712,5	< 0,0001
AF (un)	47,2	29,8	< 0,0001
BF/AF	1,18	3,85	< 0,0001

BF: componente de baixa frequência da VFC; AF: componente de alta frequência da VFC; ms^2^: milissegundos elevados ao quadrado; un: unidades normalizadas; TI: teste de inclinação.

**Figura 1:**
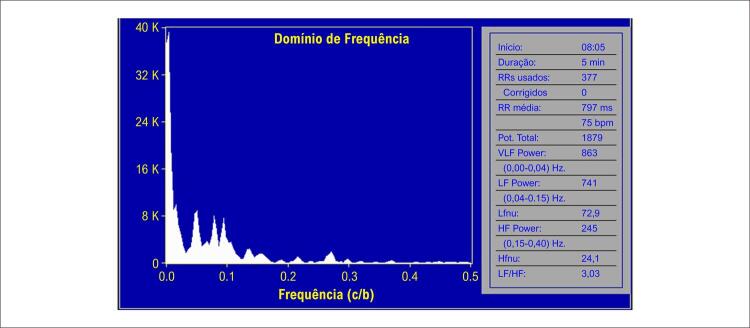
– Representação da VFC de uma paciente feminina na posição supina, com resposta vasodepressora ao teste de inclinação. LF: componente BF; HF: componente AF. Power em ms2; nu: unidades normalizadas.

Nas Figuras 1 e 2 estão demonstrados os valores da VFC na posição supina e durante o TI, aos 10 minutos, respectivamente, de uma paciente com resposta vasovagal vasodepressora.

**Figura 2:**
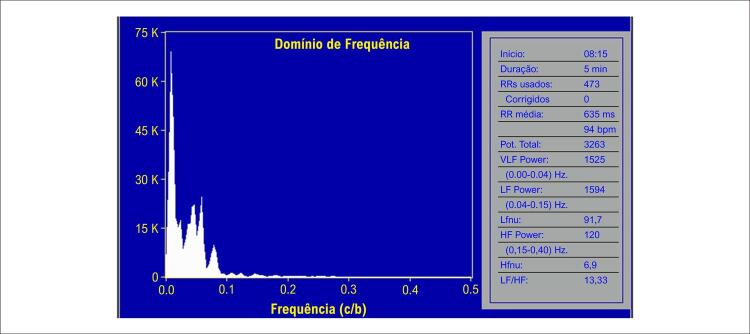
– Representação da VFC da mesma paciente referente a Figura 1 com dados aos 10 minutos do teste de inclinação, com resposta vasodepressora. Houve aumento do componente BF e da relação BF/AF e houve diminuição do componente AF em relação à posição supina.

### Associação de variáveis relacionadas ao TI

Não houve associação entre a resposta positiva ao TI e sexo, como também entre resposta vasovagal e sexo. A resposta vasovagal ocorreu em 76,3% dos casos no sexo feminino e em 65,7% dos casos no masculino (p = 0,31). A mediana de idade foi de 14,0 anos naqueles com resposta vasovagal e 12,4 naqueles com TI negativo (p = 0,04). Não houve associação entre resposta vasovagal e as variáveis: intervalo entre o último episódio de síncope e o TI, recorrência de síncope, assim como com os escores Calgary e Calgary modificado.

**Figura 3:**
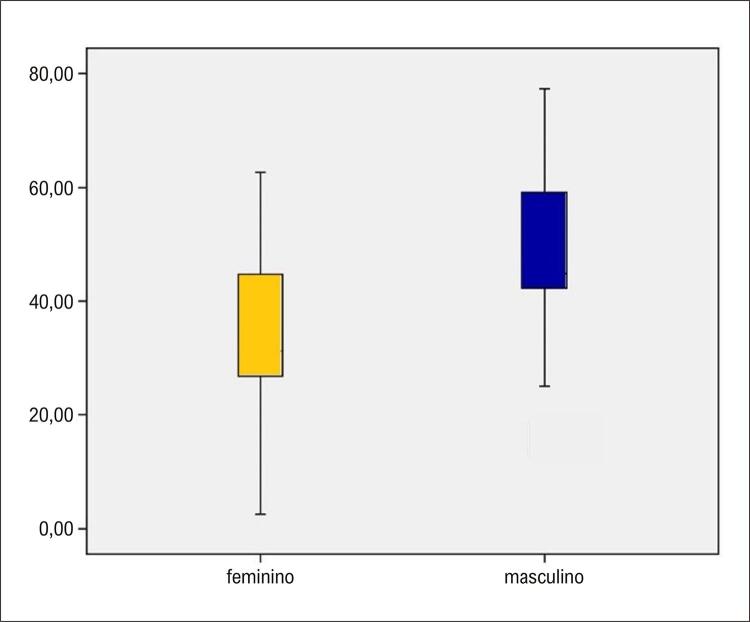
– Diagrama de caixas (boxplot) demonstrando a comparação entre os sexos em relação ao componente BF na posição supina (eixo da ordenada) para o grupo com resposta vasovagal.

No tangente à VFC, não houve diferença quanto aos componentes BF, AF, relação BF/AF e sexo, seja na posição supina ou durante o TI, independente da resposta. Todavia, considerando somente aqueles com resposta vasovagal positiva, houve diferença entre os sexos quanto ao componente BF em un, com valores de 33,6 e 47,4 para os sexos feminino e masculino, respectivamente, com p = 0,02 ( Figura 3 ).

Em relação à variável trauma físico, houve associação com o componente BF do TI com média de 32,1 un para os pacientes sem trauma e 24,3 para aqueles com trauma, com p = 0,02.

Na Figura central estão dispostos os principais resultados sobre o perfil clínico e o teste de inclinação.

**Figura Central:**
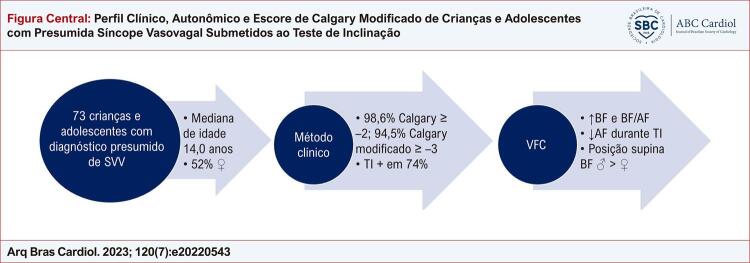
: Perfil Clínico, Autonômico e Escore de Calgary Modificado de Crianças e Adolescentes com Presumida Síncope Vasovagal Submetidos ao Teste de Inclinação

Principais resultados sobre o perfil clínico e o teste de inclinação. SVV: vasovagal; TI: teste de inclinação; VFC: variabilidade da frequência cardíaca; BF: componente de baixa frequência; AF: componente de alta frequência.

### Análise da Curva de Operação Característica (curva ROC)

**Figura 4:**
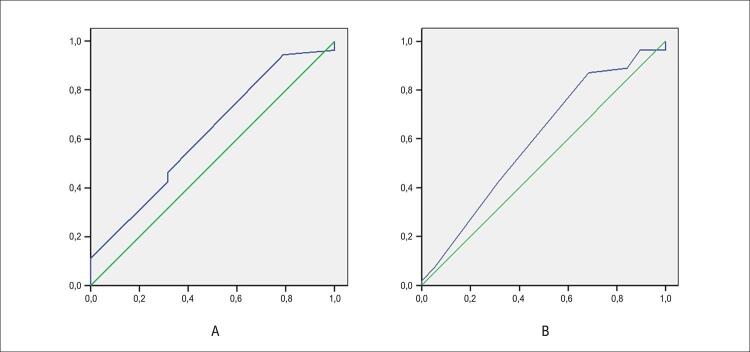
– Curva de operação característica para o escore Calgary (A) e para o escore Calgary modificado (B), considerando a resposta positiva ao TI. No eixo das ordenadas os valores de sensibilidade, e nas abscissas, o complemento da especificidade, ou seja, o valor (1-especificidade).

Aplicando-se a curva de operação característica para toda a casuística, considerando a variável estável resposta positiva, foram obtidas as áreas abaixo da curva de 0,62 e 0,60, respectivamente, para os escores de Calgary e de Calgary modificado, sem significância estatística (valores-p de 0,12 e 0,21, respectivamente) ( Figura 4 ).

**Figura 5:**
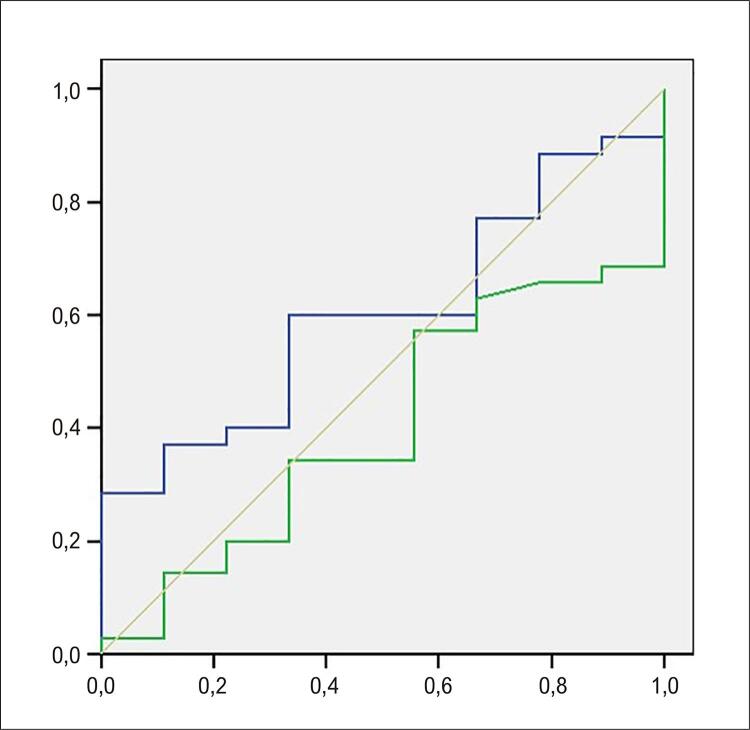
– Curva de operação característica para os componentes BF (linha azul) e AF (linha verde) na posição supina, em unidades normalizadas, considerando a resposta positiva.

Empregando a curva ROC para a mesma variável estável, com os componentes da análise espectral, foram obtidas as áreas abaixo da curva de 0,60 para o componente de BF na posição supina e 0,40 para o componente de AF na mesma posição, ambos em un. As áreas foram de 0,52, 0,43 e 0,58, respectivamente, para os componentes de BF, AF e a relação BF/AF na posição supina em ms^2^. Ao analisar esses componentes, não houve significância estatística em un, com valores-p de 0,34 e 0,36 ( Figura 5 ), e tampouco em ms^2^.

## Discussão

No presente estudo, 52% das crianças e adolescentes submetidos ao TI com diagnóstico presumido de SVV pertenciam ao sexo feminino, e 80% delas apresentaram pródromos. Os escores de Calgary e Calgary modificado foram sugestivos de SVV em 98,6% dos casos. A maioria dos pacientes apresentou TI positivo com predomínio da SVV (67%), com resposta vasodepressora. Houve maior ativação simpática no sexo masculino na resposta vasovagal. A análise da curva ROC, em relação à variável estável resposta positiva ao TI, não demonstrou significância estatística quanto aos escores e à VFC.

Quanto ao perfil clínico, a idade e a maior frequência do sexo feminino foram semelhantes ao descrito por outros estudos sobre síncope em população de crianças e adolescentes, com médias de idade de 14,6 e 15,6 anos, e proporções do sexo feminino de 59,4% e 70%.^6 , 11^

Os sintomas prodrômicos são importantes para a caracterização da causa da síncope. Na SVV clássica, esses sintomas são autonômicos como palidez, sudorese, calor, dor abdominal, náusea, vômito e visão turva.^1 , 2^ Podem ocorrer em cerca de 80% dos pacientes,^12^ em conformidade com o estudo em questão.

Segundo dados na população pediátrica, a taxa de recorrência da síncope está entre 24,7% e 38,5%,^13 , 14^ sem preferência de sexo. Em adultos, esta taxa pode ser de 60% ao se considerar pelo menos 2 episódios, e com uma média de 3,7 episódios por paciente.^12^ Em jovens, a taxa pode atingir 64%.^15^ No presente estudo, a taxa foi de 68,5% quando se considerou o número de pelo menos 3 episódios prévios, com uma mediana de 4 episódios por paciente. Esta taxa correspondeu aos episódios relatados antes do TI. Talvez por isso, essa taxa seja maior, uma vez que os pacientes não haviam recebido orientações e recomendações antes do diagnóstico da resposta pelo TI.

Em 2017, foi publicada a diretriz sobre a abordagem da síncope na população pediátrica, com a recomendação do uso do escore de Calgary modificado para distinguir SVV de outras causas de síncope. A partir de então, somente 3 estudos foram publicados sobre o tema.^16 - 18^ Foram demonstradas taxas de 95,4% e 96,3% de sensibilidade e de 67,7% e 72,7% de especificidade para o escore ≥ 2,5 para diferenciar a síncope cardíaca de SVV e de STOP, respectivamente.^16 , 17^ Para a diferenciação de SVV e epilepsia, o valor ≥ 1 apresentou sensibilidade de 92,7% e especificidade de 96,6%^6^ . Nesta pesquisa, 98,6% dos pacientes obtiveram a pontuação ≥ -2 para o escore de Calgary e 94,5% pontuação ≥ -3 na escala de Calgary modificado. Em razão disso, o TI foi positivo em 74%, considerando as respostas reflexas e a curva ROC não apresentou significância estatística para os escores. Ou seja, a população do presente estudo foi homogênea quanto ao diagnóstico de SVV, sem incluir pacientes com síncope cardíaca ou com epilepsia. E a taxa de positividade do TI apresentou valores entre as taxas descritas na literatura na população pediátrica, de 65%, em 150 pacientes,^13^ e de 95%, em 49 pacientes.^6^

No que tange ao padrão de resposta vasovagal ao TI, a maioria apresentou resposta vasodepressora (até 77,4%), seguida da resposta mista,^19 , 20^ similar ao estudo em questão. Todavia, há outros estudos com casuística menor que demonstraram resposta mista predominante, com taxas de 74,3% e 49,4%.^6 , 9^ A média do tempo de resposta vasovagal do presente estudo também foi semelhante ao da literatura, cujos valores foram de 13 e 15 min.^14 , 21^

A avaliação da VFC tem sido utilizada como ferramenta simples e não invasiva em pacientes adultos com quadro de SVV durante o TI, representando um importante marcador quantitativo do balanço autonômico, com sensibilidade de 95% para predição da síncope.^22^ Portanto, os testes autonômicos podem ser adjuvantes nesse diagnóstico^2^ , uma vez que o sistema nervoso autônomo está implicado na fisiopatologia da SVV. Na população pediátrica, há alguns estudos sobre essa ferramenta com registros de curta duração para análise espectral durante o TI^7 , 23 - 27^ e outros para diferenciação entre SVV e STOP, com gravações pelo sistema Holter de 24 horas.^28 , 29^

No presente estudo, durante o TI, em toda a casuística, houve um aumento expressivo do componente de BF, da relação BF/AF e da diminuição de AF, quando os valores foram comparados com os da posição supina. Esses dados são consistentes com os reportados por outros estudos na população pediátrica^7 , 23 - 27 , 30 , 31^ que sugeriram hipóteses como ineficiência do tônus simpático diante o ortostatismo, falha no reflexo barorreceptor após a inclinação, instabilidade hemodinâmica com diminuição do débito cardíaco e/ou da resistência vascular periférica, fatores que resultaram em síncope nos predisponentes. Entretanto, diferente dos estudos prévios com cerca de 24 a 51 crianças e adolescentes com SVV,^23 - 25 , 27^ a VFC por meio da curva ROC não permitiu discriminar aqueles com resposta positiva ao TI neste estudo, uma vez que não foi incluído grupo controle (sem histórico de SVV), em 98,6% da casuística os escores de Calgary e Calgary modificado foram sugestivos de SVV e o TI foi positivo em 74%. Miranda et al.,^32^ com uma população selecionada, também não reportaram diferença significante referente aos componentes da VFC no domínio da frequência entre o grupo com TI com resposta cardioinibitória (40 pacientes) e o grupo controle com TI negativo (24 pacientes) durante a inclinação, corroborando o resultado deste estudo. A idade mínima foi de 14 anos, porém a média de idade foi de 36,2 anos no estudo citado.^32^

Referente à comparação da variável clínica sexo, com os componentes da análise espectral da FC no grupo com resposta vasovagal positiva, houve diferença quanto ao componente BF em unidades normalizadas, sendo que os participantes do sexo masculino apresentaram uma maior expressão deste componente na posição supina em relação àqueles do sexo feminino. Um estudo anterior e uma meta-análise com um total acumulado de 292.247 participantes,^33 , 34^ sendo a maioria adultos, todavia com inclusão de participantes de 5 meses de idade e outros a partir de 5 anos, confirmaram esse resultado. Os autores concluíram que o coração masculino é influenciado predominantemente por atividade simpática, predispondo-o ao risco de doenças cardiovasculares. Em crianças, há um aumento colinérgico e uma diminuição da atividade adrenérgica, refletindo um amadurecimento da função autonômica, com um período de transição na adolescência, entre as idades de 12 e 14 anos.^35 - 37^ Porém, em razão da puberdade chegar mais cedo nas meninas, pode haver diferenças autonômicas quanto aos sexos também na infância, conforme a faixa etária. Um estudo com 1036 crianças, sem doenças cardiovasculares e com média de idade de 10,2 anos, demonstrou uma FC mais elevada e maior relação BF/AF entre as meninas, o que foi atribuído à sua puberdade. Em contrapartida, outro estudo, com 321 indivíduos saudáveis, com idades entre 6 e 13 anos, apesar de ter demonstrado aumento da FC e menor VFC em meninas, evidenciou que o sexo não foi determinante na VFC na análise multivariada.^38^ Portanto, há variações da VFC relacionadas à idade e ao sexo. Em crianças saudáveis abaixo dos 12 anos de idade, parece não haver influência do sexo; contudo, a partir dessa idade, com as alterações descritas previamente no SNA, há uma influência do sexo, com ativação do eixo hipotálamo-hipófise-adrenal no sexo masculino.^36^ Toda esta explanação pode explicar os achados do presente estudo, realçando que todas as crianças e adolescentes apresentavam o diagnóstico presumido de SVV e com idade variando de 6 a 18 anos.

Outro aspecto deste estudo referiu-se à importância do método clínico, pedra angular da medicina. A abordagem clínica integral permite o diagnóstico de SVV e o TI também contribui para confirmar o diagnóstico e o padrão de resposta, evitando a realização de outros exames que seriam desnecessários.^1 , 2 , 5^ Entretanto, na prática clínica, muitos exames complementares, além do ECG, são feitos por solicitação dos médicos assistentes, como neste estudo, evidenciando a importância da aderência às diretrizes e do treinamento da equipe para a implementação delas.^13^

O TI é um exame seguro, considerado uma estratégia de diagnóstico com classe de recomendação II A. O uso de agentes farmacológicos, após a fase passiva do TI, aumenta a sua sensibilidade, contudo com diminuição da sua especificidade, além da possibilidade de raras complicações.^1 , 5 , 39^ Na população pediátrica, a fase farmacológica, seja com isoproterenol ou nitroglicerina, foi utilizada em alguns estudos.^6 , 20 , 40 - 42^ No entanto, a nitroglicerina pode produzir sintomas vasovagais mais prolongados, com menor sensibilidade, induzindo uma resposta vasodepressora, não sendo recomendado seu uso habitual.^41 - 43^ Neste presente estudo, não foi utilizado nenhum desses agentes, não obstante o acesso venoso periférico eletivo, o qual foi feito seguido de repouso na posição supina durante 20 minutos, como preconizado pelas diretrizes.^1 , 2^

### Limitações

A principal limitação deste estudo foi a falta de um grupo controle de pacientes, que impediu a comparação entre aqueles com SVV e os saudáveis. Além disso, o TI não foi realizado com provocação farmacológica, o que pode ter resultado em uma porcentagem menor de resposta positiva. Não foi utilizada pletismografia digital para aferir os níveis pressóricos, constituindo isso também uma limitação.

## Conclusões

Neste estudo, a maioria das crianças e adolescentes com diagnóstico presumido de SVV apresentou um cenário clínico típico, com escore de Calgary ≥ -2. O padrão de resposta predominante ao TI foi a vasodepressora. Houve maior ativação simpática na posição supina nos indivíduos do sexo masculino. Os escores de Calgary e a ativação simpática não permitiram predizer a resposta ao TI.
