# Supplementary material for: Perfil Clínico, Autonômico e Escore de Calgary Modificado de Crianças e Adolescentes com Presumida Síncope Vasovagal Submetidos ao Teste de Inclinação
Source: Arq Bras Cardiol. 2023 Jul 27;120(7):e20220543. [Article in Portuguese] doi: 10.36660/abc.20220543 (PMC10382152; doi:10.36660/abc.20220543)
Supplement: Translation

# Translation: Clinical and Autonomic Profile, and Modified Calgary Score for Children and Adolescents with Presumed Vasovagal Syncope Submitted to the Tilt Test

**Authors:** Pamela Michelle Leite Oliveira, Rose Mary Ferreira Lisboa da Silva, Henrique de Assis Fonseca Tonelli, Zilda Maria Alves Meira, Cleonice de Carvalho Coelho Mota

**Affiliations:** 1 Brazil Universidade Federal de Minas Gerais, Belo Horizonte, MG – Brazil; 2 Brazil Hospital das Clínicas da Universidade Federal de Minas Gerais, Belo Horizonte, MG – Brazil

**Keywords:** Syncope, Vasovagal, Heart Rate, Tilt-Table Test, Child, Adolescent

## Abstract

**Background:**

In the pediatric population, syncope is mainly from vasovagal (VVS) origin. Its evaluation must be done by clinical methods, and the tilt test (TT) can contribute to the diagnosis.

**Objectives:**

To analyze the clinical profile, Calgary and modified Calgary scores, response to TT and heart rate variability (HRV) of patients aged ≤ 18 years with presumed VVS. To compare the variables between patients with positive and negative responses to TT.

**Method:**

Observational and prospective study, with 73 patients aged between 6 and 18 years, submitted to clinical evaluation and calculation of scores without previous knowledge of the TT. It was done at 70º under monitoring for HRV analysis. P-value < 0.05 was the statistical significance criterion.

**Results:**

Median age was 14.0 years; 52% of participants were female, 72 had Calgary ≥ -2 (mean 1.80), and 69 had modified Calgary ≥ -3 (mean 1.38). Prodromes were observed in 59 patients, recurrence in 50 and trauma in 19. The response to TT was positive in 54 participants (49 vasovagal, with 39 vasodepressor responses), with an increase in the low frequency (LF) component and a decrease in the high frequency (HF) component (p < 0,0001). In the supine position, LF was 33.6 in females and 47.4 in normalized units for males (p = 0.02). When applying the operating characteristic curve for positive TT, there was no statistical significance for HRV and scores.

**Conclusion:**

Most children and adolescents with a presumed diagnosis of VVS presented a typical clinical scenario, with a Calgary score ≥ -2, and a predominant vasodepressor response to TT. Greater sympathetic activation was observed in the supine position in males. Calgary scores and sympathetic activation did not predict the response to TT.

## Introduction

A syncope is characterized by a transient loss of consciousness and postural tone, resulting from transient cerebral hypoperfusion, having a sudden onset, short duration and spontaneous and complete recovery.^1 , 2^ Pediatric syncope has numerous causes, with the main one (about 80% of cases) being from vasovagal (VVS) origin.^3^

Syncope evaluation is mostly done through anamnesis, physical examination, blood pressure (BP) measurement, in supine and orthostatic positions, and electrocardiogram (ECG) imaging.^1 , 2^ An essential instrument for the propaedeutics of syncope in children and adolescents is the modified Calgary score, used to perform the differential diagnosis in patients with VVS and other causes of syncope.^4^

Following an appropriate clinical approach, the tilt test (TT) is a valuable and useful tool that can avoid unnecessary laboratory tests in patients with no evidence of heart disease and with a clinical history suggestive of VVS, in whom this diagnosis has not yet been made and/or when the elucidation of the response pattern to TT is required.^5^ In the pediatric population, its sensitivity and specificity can be 76.6% and 86.7%, respectively.^6^ TT contributes to differential diagnosis between VVS and its other subtypes: vasodepressor; cardioinhibitory; mixed; postural orthostatic tachycardia syndrome (POTS); and orthostatic hypotension (OH), as well as the distinction between syncope and epilepsy.

Autonomic nervous system (ANS) disorders are well-known to be implicated in the pathophysiology of VVS. Under provocative conditions, such as during TT,^7^ patients with this condition have increased baseline sympathetic activity and decreased parasympathetic activity. Thus, the ANS assessment is important in understanding syncope and conducting propaedeutics for these patients.

Due to the scarce literature on VVS, TT and ANS evaluation in the pediatric population, this study aimed to analyze the clinical profile, the Calgary and modified Calgary scores; its association with the occurrence of pre-syncope or syncope with TT in children and adolescents with a history of VVS, and to evaluate heart rate variability (HRV) by pre-TT spectral analysis, in the supine position, and also during TT.

## Method

This was a cohort, observational, cross-sectional and prospective study carried out from February 2016 to March 2020. The research was approved by the local research ethics committee, and the participants and/or their legal guardians were asked to sign the assent and/or consent form. The sample consisted of 73 patients. Those aged 6 years or older, aged up to 18 years, with a presumed diagnosis of VVS and without structural heart disease, as well as those without channelopathies (with a normal ECG, no personal and/or family history of syncope or arrhythmia-related sudden cardiac death). Patients with cardiac, neurological, psychiatric and chronic diseases, arrhythmias, users of drug products with potential interference on heart conduction velocity, heart rate (HR) or BP, as well as those on artificial pacemakers and any other conditions that could hinder cardiac rhythm, were excluded from the study.

The patients were submitted to clinical evaluation, and their Calgary and modified Calgary scores were calculated ( Table 1 )^4 , 8^ by a researcher blind to the TT results. The TTs were performed in the morning shift, in a suitable room and under low lighting. Patients had fasted for 6 hours and had elective peripheral venous access, remaining in the supine position, at rest, for 20 minutes. The tilting table had a footrest and allowed variations in the inclination angle at 10-degree intervals. The tests were performed at an angle of 70 degrees and for a time limit of 30 minutes in case there was no positive response. Patients were monitored with continuous 12-lead ECG and non-invasive BP measurement on the arm (recorded every 3 minutes), from the resting phase until the end of recovery, according to standardized TT equipment and techniques.^9^ Monitoring was also performed using Holter Cardiosmart to assess HRV, with the patient in the supine position at the first 10 minutes or last 5 minutes of the TT before the syncopal event, in case of a positive response. Analysis was performed after extrasystole correction, with pauses and interference by an experienced cardiologist, using the Fourier transformation, and obtaining the absolute power values in milliseconds squared (ms^2^) and the values in normalized units (nu) of the low-frequency (LF) and high-frequency (HF) components, and the LF/HF ratio. According to the literature, the HF component is mainly led by vagal activity, and the LF/HF ratio reflects the sympathovagal balance.^10^

**Table 1:** – Modified Calgary score items ^4^

Question	Score (for positive answers)
Is there a bifascicular block to the patient’s ECG, or a history of asystole or supraventricular tachycardia?	- 5
At times, have bystanders noted that the patient turns blue during a syncope?	- 4
Did the syncopal episodes start when the patient was ≤ 5 years old?	- 3
Does the patient remember anything about being unconscious?	- 2
Does the patient feel faint when sitting or standing for a prolonged period of time?	+ 1
Does the patient sweat before a syncopal episode?	+ 2
Does the patient feel faint when in pain or medical settings?	+ 3

ECG: electrocardiogram. NOTE: The differences in the Calgary score ^8^ are observed in question 1 (which also includes diabetes) and in question 3 (which considers the onset of syncopal episodes at 35 years or older).

### Statistical analysis

Statistical analysis was performed using the SPSS software for Windows, version 14.0 (SPSS, Inc., Chicago, Illinois). Qualitative variables were presented using frequencies, and continuous variables, using mean and standard deviation (for variables with normal distribution), and median and interquartile ranges (for variables without normal distribution), according to the Kolmogorov-Smirnov test.

Comparison between groups was performed as follows: Student’s t test (unpaired) for continuous variables with normal distribution or non-parametric Mann-Whitney test for variables with non-Gaussian distribution; chi-square test for qualitative variables (or Fisher’s exact test, when appropriate). The Wilcoxon test was used to compare paired variables. The operating characteristic curve was applied to assess the sensitivity and specificity of the Calgary and modified Calgary scores, and that of the spectral analysis components, by considering the positive response to the TT. P-value < 0.05 was the statistical significance criterion.

## Results

### General characteristics of casuistry

The median age of patients was 14.0 years (interquartile range – Q1-Q3: 11.0 - 16.0), ranging from 6 to 18 years, with 38 female children (52.0%). Fifty-nine patients had prodromes (80.8%), with 19 related to physical trauma resulting from VVS. The most commonly reported prodromal symptoms were asthenia, pale skin, cold sweating and blurred vision. There was a history of recurrence (equal to or greater than three syncopal episodes) in 50 patients. Seventy-two had a Calgary ≥ -2, with a mean of 1.80, and 69 had a modified Calgary ≥ -3, with a mean of 1.38.

The median time of symptoms’ evolution was 19.0 months (4.5 – 55.0). The median interval between the last syncopal episode and the TT was 41.0 days. As for the number of episodes, the median was 4.0 (2.0 – 8.0).

Regarding the complementary exams performed before the TT, 42 patients underwent echocardiography, 31 underwent Holter monitoring, 13 underwent exercise stress tests, 36 underwent clinical pathology exams, and 28 were evaluated by neurology.

### Association of clinical variables

Concerning the sex variable, there was a difference in age, with a median of 15.0 years (13.7 – 16.0) in females and 13.0 (10.0 – 15.0) in males, p = 0.02. There was no association with the variables of time of evolution, the proportion of prodromes, trauma, number of syncopal episodes, the interval between the last episode and the day of the TT, and the Calgary and modified Calgary scores. Both sexes underwent the same proportion of complementary exams.

As for physical injuries, there was an association with the modified Calgary score only, with a mean of 0.7 in the group with physical injury and 1.6 in the group without physical injury (p = 0.01).

### TT-Related variables

The response to TT was positive in 54 patients (74%), namely: vasovagal response in 49, OH in 3 patients and POTS in 2. Concerning the vasovagal-type response, 39 had a vasodepressor response, 7 had a cardioinhibitory response, and 3 had a mixed response. The mean time to vasovagal response to TT was 13.9 ± 9.4 minutes, ranging from 1.25 to 33 minutes.

The mean drop in systolic BP during TT was 110 mmHg in patients with a vasodepressor response, and the mean HR drop was 61 bpm in patients with a cardioinhibitory response. Among patients with a positive response to the TT, 9 did not show complete spontaneous recovery after returning to the supine position, requiring medical intervention with atropine (maximum dose of 1.0 mg) and/or saline solution 0.9% (maximum 300ml) administration.

### Assessment of heart rate variability through spectral analysis

When comparing the HRV components in the supine position and at 10 minutes of inclination using the Wilcoxon test in the entire casuistry, the mean values of the LF and HF components and the relationship between them were obtained, as shown in Table 2 .

**Table 2:** – Spectral analysis of the patients’ heart rate in the supine position at the 10th minute of the tilt test

Variables	Supine position	10th minute of TT	p-value
LF (ms^2^)	1819.4	1326.6	0.05
LF (un)	44.0	61.6	< 0.0001
HF (ms^2^)	2735.9	712.5	< 0.0001
HF (un)	47.2	29.8	< 0.0001
LF/HF	1.18	3.85	< 0.0001

LF: HRV low-frequency component; HF: HRV high-frequency component; ms^2^: milliseconds squared; nu: normalized units; TT: tilt test.

Figures 1 and 2 show the HRV values in the supine position and at the 10th minute of the TT, respectively, of a patient with a vasovagal vasodepressor response.

**Figure 1:** – Representation of the HRV of a female patient in the supine position, with a vasodepressor response to the tilt test. LF: LF component; HF: HF component. Power in ms2; nu: normalized units.

**Figure 2:** – HRV representation of the same patient referred to in Figure 1 with data taken at the 10th minute of the tilt test, with a vasodepressor response. There was an increase in the LF component and the LF/HF ratio, and a decrease in the HF component in relation to the supine position.

### Association of TT-Related variables

No association was observed between the positive response to TT and sex nor between the vasovagal response and sex. Vasovagal response occurred in 76.3% of cases in females and 65.7% of cases in males (p = 0.31). The median age was 14.0 years in those with a vasovagal response and 12.4 in those with negative TT (p = 0.04). There was no association between vasovagal response and the following variables: the interval between the last syncopal episode and TT, syncope recurrence, and Calgary and modified Calgary scores.

Regarding HRV values, there was no difference regarding the LF, HF, LF/HF ratio and sex, either in the supine position or during TT, regardless of the response. However, considering only those with a positive vasovagal response, there was a difference between sexes regarding the LF component in nu, with values of 33.6 and 47.4 for females and males, respectively, and p = 0.02 ( Figure 3 ).

**Figure 3:** – Boxplot showing the comparison between sexes concerning the LF component in the supine position (ordinate axis) for the group with a vasovagal response.

Regarding the physical trauma variable, there was an association with the LF component of the TT, with a mean of 32.1 nu for patients without trauma and 24.3 for those with trauma, and p = 0.02.

The main results of the clinical profile and the tilt test are displayed in the central illustration .

**Central Illustration:** : Clinical and Autonomic Profile, and Modified Calgary Score for Children and Adolescents with Presumed Vasovagal Syncope Submitted to the Tilt Test

Main results on clinical profile and tilt test. VVS: vasovagal; TT: tilt test; HRV: heart rate variability; LF: low-frequency component; HF: high-frequency component; HRV: heart rate variability.

### Analysis of the Receiver Operating Characteristic Curve (ROC curve)

When applying the operating characteristic curve for the entire casuistry and considering the stable positive response variable, areas under the curve of 0.62 and 0.60, respectively, were obtained for the Calgary and modified Calgary scores, with no statistical significance (p-values = 0.12 and 0.21, respectively) ( Figure 4 ).

**Figure 4:** – Operating characteristic curve for the Calgary score (A) and modified Calgary (B) score, considering the positive response to TT. On the ordinate axis, the sensitivity values, and on the abscissas axis, the complement of the specificity, that is, the value (1-specificity).

Using the ROC curve for the same stable variable with the components of the spectral analysis, the areas under the curve of 0.60 for the LF component in the supine position and 0.40 for the HF component in the same position were obtained, both in nu. The areas were 0.52, 0.43 and 0.58, respectively, for the LF and HF components and the LF/HF ratio in the supine position in ms^2^. Upon analysis of these components, no statistical significance was observed in nu, with p-values of 0.34 and 0.36 ( Figure 5 ), nor in ms^2^.

**Figure 5:** – Operating characteristic curve for the LF (blue line) and HF (green line) components in the supine position, in normalized units, considering the positive response.

## Discussion

In this study, 52% of children and adolescents undergoing TT with a presumed VVS diagnosis were female, and 80% had prodromes. Calgary and modified Calgary scores were suggestive of VVS in 98.6% of cases. Most patients had a positive TT with a predominance of VVS (67%) with vasodepressor response. There was greater sympathetic activation in males in the vasovagal response. The ROC curve analysis, concerning the stable variable of positive response to TT, did not demonstrate statistical significance regarding the scores and HRV.

As for the clinical profile, the age and higher frequency of females were similar to that described by other studies on syncope in children and adolescents, with mean ages of 14.6 and 15.6 years, and proportions of females of 59.4% and 70%.^6 , 11^

Prodromal symptoms are important for identifying the cause of syncope. In classic VVS, these autonomic symptoms include paleness, sweating, heat, abdominal pain, nausea, vomiting and blurred vision.^1 , 2^ According to the study, they may occur in approximately 80% of patients.^12^

According to data on the pediatric population, the syncope recurrence rate is between 24.7% and 38.5%,^13 , 14^ without sex-related prevalence. This rate can be as high as 60% in adults when considering at least two episodes, with an average of 3.7 episodes per patient.^12^ In young people, this rate can reach 64%.^15^ This study showed a 68.5% rate when considering the number of at least 3 previous episodes, with a median of 4 episodes per patient. This rate corresponded to pre-TT episodes, which may be the reason for this increased rate since the patients had not been oriented before the diagnosis of the response to the TT.

In 2017, a syncope management guideline for the pediatric population was published, with the recommendation to use the modified Calgary score to distinguish VVS from other causes of syncope. Since then, only three studies have been published on the subject.^16 - 18^ Sensitivity rates of 95.4% and 96.3% and specificity rates of 67.7% and 72.7% were demonstrated for a score ≥ 2.5 to differentiate cardiac syncope from VVS and POTS, respectively.^16 , 17^ In order to differentiate between VVS and epilepsy, a value ≥ 1 showed a sensitivity of 92.7% and specificity of 96.6%.^6^ In this research, 98.6% of patients scored ≥ -2 on the Calgary score and 94.5% scored ≥ -3 on the modified Calgary scale. As a result, the TT was positive in 74%, considering the reflex responses and the ROC curve and no statistical significance was shown for the scores. That is, the population of this study was homogeneous regarding VVS diagnosis, when excluding patients with cardiac syncope or epilepsy, and the positive TT rate showed values between the rates described in the literature for the pediatric population, of 65%, in 150 patients,^13^ and 95%, in 49 patients.^6^

Regarding the vasovagal response pattern to TT, the majority showed a vasodepressor response (up to 77.4%), followed by a mixed response,^19 , 20^ similar to said study. However, other studies with a smaller sample size showed a predominant mixed response, with rates of 74.3% and 49.4%.^6 , 9^ In the present study, the mean vasovagal response time was also similar to that in the literature, whose values were 13 and 15 minutes.^14 , 21^

The HRV evaluation has been used as a simple and non-invasive tool in adult patients with VVS during the TT. It represents an important quantitative marker of autonomic balance, with a 95% sensitivity for syncope prediction.^22^ Therefore, autonomic tests can help in this diagnosis^2^ since the autonomic nervous system is implicated in the pathophysiology of VVS. In the pediatric population, some studies on this tool have short-term recordings for spectral analysis during TT^7 , 23 - 27^ and others for differentiating between VVS and POTS, with recordings by the 24-hour Holter system.^28 , 29^

In the present study, during TT, in the entire casuistry, there was a significant increase in the LF component, the LF/HF ratio and the HF reduction, when the values were compared with those in the supine position. These data are consistent with those reported by other studies in the pediatric population^7 , 23 - 27 , 30 , 31^ which have suggested hypotheses such as ineffective sympathetic tonus in the face of orthostatism, baroreceptor reflex failure after bending over, hemodynamic instability with decreased cardiac output and/or peripheral vascular resistance, factors that resulted in syncope in predisposing individuals. However, unlike previous studies with 24 to 51 children and adolescents with VVS,^23 - 25 , 27^ the HRV using the ROC curve did not identify those with a positive response to TT in this study since a control group was not included (without a history of VVS), in 98.6% of the sample, the Calgary and modified Calgary scores were suggestive of VVS and the TT was positive in 74%. Miranda et al.,^32^ with a selected population, also did not report a significant difference regarding the HRV components in the frequency domain between the group with a cardioinhibitory response to TT (40 patients) and the control group with a negative response to TT (24 patients) during inclination, corroborating the result of this study. The minimum age was 14 years, but the mean age was 36.2 years in the referenced study.^32^

Regarding the comparison of the clinical variable sex with the components of the spectral analysis of the HR in the group with a positive vasovagal response, there was a difference regarding the LF component in normalized units, and male participants showed a greater expression of this component in the supine position when compared to females. A previous study and a meta-analysis with a cumulative total of 292,247 participants,^33 , 34^ the majority being adults; however, the inclusion of participants aged 5 months to 5 years and older confirmed this result. The authors concluded that the male heart is predominantly influenced by sympathetic activity, with an increased predisposition to the risk of cardiovascular diseases. In children, there is increased cholinergic and decreased adrenergic activity, reflecting a maturation of the autonomic function, with a transition period between the ages of 12 and 14.^35 - 37^ However, as puberty onset is usually earlier for girls, there may be autonomic differences between sexes in childhood as well, according to the age group. A study with 1036 children without cardiovascular diseases, with a mean age of 10.2 years, demonstrated a higher HR and a higher LF/HF ratio among girls, which was attributed to their puberty status. On the other hand, another study, with 321 healthy individuals aged between 6 and 13 years, showed that sex was not a determinant factor in HRV in the multivariate analysis,^38^ despite having demonstrated increased HR and lower HRV in girls. Therefore, there are variations in HRV related to age and sex. In healthy children under 12 years of age, there appears to be no influence of sex; however, from that age onwards, with the previously described ANS changes, an influence of sex is observed, with activation of the hypothalamic-pituitary-adrenal axis in males.^36^ All of those can explain the findings of this study, emphasizing that all children and adolescents had the presumed diagnosis of VVS and were aged between 6 and 18 years.

Another aspect of this study referred to the importance of the clinical method, the cornerstone of medicine. The integral clinical approach allows the diagnosis of VVS, and the TT also contributes to confirming the diagnosis and the response pattern, thus avoiding further unnecessary examinations.^1 , 2 , 5^ However, in clinical practice, many complementary exams, in addition to the ECG, are performed upon the attending physician’s request, as in this study, which evidences the importance of adherence to the guidelines and qualification of the team for their implementation.^13^

The tilt test is safe and considered a diagnostic strategy with recommendation class II A. The use of pharmacological agents after the passive phase of TT increases its sensitivity; however, with a decrease in its specificity, in addition to the possibility of rare complications.^1 , 5 , 39^ In the pediatric population, the pharmacological phase, either with isoproterenol or nitroglycerin, was used in some studies.^6 , 20 , 40 - 42^ However, nitroglycerin can produce prolonged vasovagal symptoms with less sensitivity, inducing a vasodepressor response, and it is not usually recommended.^41 - 43^ In this study, none of these agents were used, despite the elective peripheral venous access, which was performed followed by rest in the supine position for 20 minutes, as recommended by the guidelines.^1 , 2^

### Limitations

The main limitation of this study was the lack of a control group of patients, which hindered the comparison between those with VVS and healthy people. Moreover, the TT was not performed under pharmacological provocation, which may have resulted in a lower positive response rate. Digital plethysmography was not used to measure blood pressure levels, which is also a limitation.

## Conclusion

In this study, most children and adolescents with a presumed diagnosis of VVS presented a typical clinical scenario, with a Calgary score ≥ -2. The predominant response pattern to TT was vasodepressor. Greater sympathetic activation was observed in the supine position in males. Calgary scores and sympathetic activation did not predict the response to TT.
